# An improved *Lodderomyces elongisporus* NRRL YB-4239 genome assembly substantiated by its electrophoretic karyotype

**DOI:** 10.1128/MRA.00596-23

**Published:** 2023-09-29

**Authors:** Lois L. Hoyer, Elizabeth K. Hogan, Brian A. Freeman, Kimberly K. O. Walden, Alvaro G. Hernández

**Affiliations:** 1 Department of Pathobiology, College of Veterinary Medicine, University of Illinois Urbana-Champaign, Urbana, Illinois, USA; 2 Roy. J. Carver Biotechnology Center, University of Illinois Urbana-Champaign, Urbana, Illinois, USA; 3 Department of Mathematics and Computational Sciences, Millikin University, Decatur, Illinois, USA; University of California, Riverside, California, USA

**Keywords:** yeast, genome sequence, karyotype

## Abstract

Pacific Biosciences long-read sequencing was used to improve the genome assembly for *Lodderomyces elongisporus* strain NRRL YB-4239 (ATCC 11503). The new assembly included eight chromosomes that were substantiated by the electrophoretic karyotype. The nuclear genome was 16.1 Mb (37.2% GC) with 5,740 genes predicted.

## ANNOUNCEMENT

The diploid yeast *Lodderomyces elongisporus* (phylum Ascomycota, class Saccharomycetes, family Debaryomycetaceae) is closely related to *Candida parapsilosis* and isolated as the cause of human disease (1). The GenBank RefSeq assembly [ASM14968v1; (2)] contains 145 contigs on 28 scaffolds. We used Pacific Biosciences (PacBio) long-read sequencing to improve the genome assembly and substantiated results with the electrophoretic karyotype.

The *L. elongisporus* type strain (NRRL YB-4239, ATCC 11503) was purchased from the American Type Culture Collection (Manassas, VA) and grown on YPD agar (per liter: 10 g yeast extract, 20 g peptone, 20 g dextrose, and 20 g Bacto Agar). A liquid YPD culture was inoculated with an isolated colony and then incubated for 16 h at 30°C with 200 rpm shaking. Genomic DNA was released using Zymolyase; the preparation was extracted with phenol/chloroform and then precipitated with isopropanol (3).

Genomic DNA was sheared with a Megaruptor 3 system (Diagenode) to an average length of 13 kb. Fragments of 3 to 50 kb were selected using a BluePippin system with a 0.75% gel cassette and DNA marker S1 (Sage Science) and then converted to a sequencing library using the SMRTbell Express Template Prep Kit 2.0 (Pacific Biosciences). The library was sequenced on a single-molecule real-time (SMRT) cell 8M on a PacBio Sequel IIe system using a Sequel II Binding Kit 2.2, the circular consensus sequencing (CCS) mode, and a 30-h movie time. CCS and demultiplexing analysis used SMRT Link v10.0 (ccs -min-passes 3 -min-rq 0.99; lima -hifi-preset SYMMETRIC -split-bam-named -peek-guess). The data set had 781,559 reads (mean length = 13,294 bases; N_50_ = 13,593 bases).

Reads >20 kb were selected using filtlong v0.2.1 (4), reducing the sequence coverage from 645× to 25×. Length-filtered reads were assembled with hifiasm v0.16.1 using default parameters (5). The resulting primary contigs were used for further analysis and deposited into NCBI. Primary contig orientation was compared to scaffolds in ASM14968v1 [MUMmer v4.0.0beta2; nucmer; show-coords -rcl; show-diff -f -r; (6)]. Four contigs were reverse complemented [SeqKit v2.0.0; seq -r -p; (7)] to match gene-numbering schemes in the representative assembly.

**Fig 1:**
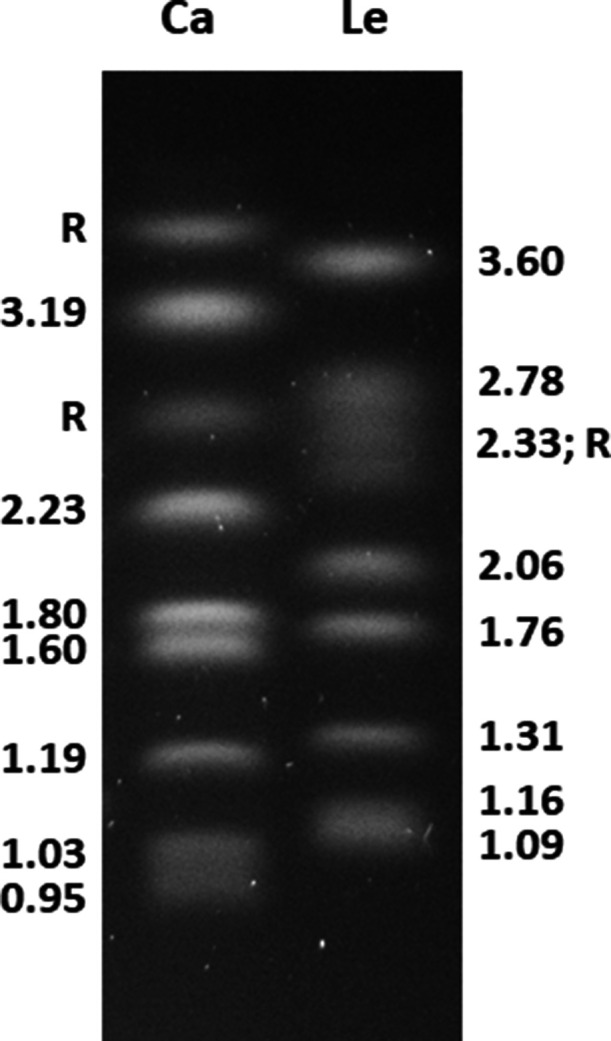
Karyotype of *Candida albicans* (Ca; left; size standard) and *L. elongisporus* (Le; right) on an ethidium-bromide-stained agarose gel. Chromosome sizes (in megabases) were placed at the side of each lane. Both species are diploid (2). *C. albicans* was used as a size standard because of its well-characterized genome assembly (10). For each species, yeast cells were embedded in agarose and then digested away leaving intact chromosome-sized DNA that was separated by clamped homogeneous electric field electrophoresis (11). Chromosomes labeled “R” have tandemly repeated units of ribosomal DNA (rDNA) that expand and contract, leading to size heterogeneity (10). The eight *L. elongisporus* contigs derived from the new genome assembly corresponded in size to the chromosomes visible on the karyotype.

The new genome assembly had eight gapless contigs matching the chromosome sizes on the *L. elongisporus* NRRL YB-4239 karyotype (Fig. 1). A 24-bp repeated motif (5′-AAGGATGCACTTGAAACTCGGTGT-3′) was located at both ends of each contig and matched previously proposed consensus telomeric sequences (2). Chromosome 8 had 12,416 bp of rDNA sequences appended to the telomeric repeats on its left end, suggesting that *L. elongisporus* may add rDNA to aging telomeres as described for other eukaryotic species (8); telomeric rDNA was trimmed from the final assembly. The percent complete and single-copy BUSCOs [BUSCO v.5.3.2; (9)] were 93.3% (fungi_odb10), 92.3% (ascomycota_odb10), and 98.6% (saccharomycetes_odb10).

The hifiasm primary contigs were cleaned, sorted, and masked using funannotate v1.8.13 with default parameters (12). To aid the funannotate “predict” program, *L. elongisporus* RNA-Seq reads [SRR3721295, SRR3721296, SRR11856477, SRR11856478; (13)] were assembled into a transcriptome using Trinity v2.14.0 [(14); --SS_lib_type RF]. The predicted coding sequences were translated with Translation Table 12 using EMBOSS Transeq (15, 16). The Table 12-translated protein sequences were analyzed using InterProScan v.5.56-89.0 (17) and eggNOG-mapper web v2.1.9 (18, 19); resulting files were included with the input for funannotate “annotate.”

The PacBio genome assembly (GCA_030384655.1) reduced contig number from 145 to 8, closed 117 gaps, and added over 0.6 Mb of new sequence distributed across all eight chromosomes (Table 1). The number of predicted genes was nearly identical to the RefSeq assembly.

**TABLE 1:** *L. elongisporus* genome assembly statistics comparison^*a*^

	Assembly accession numbers		
Feature	GCA_000149685.1 ASM14968v1	GCA_013620985.1 ASM1362098v1	GCA_030384665.1 ASM3038466v1
Total ungapped length (bp)	15,460,620	16,382,369	16,086,460
No. of contigs	145	53	8
Contig N_50_ (bp)	261,145	742,134	2,328,861
No. of scaffolds	28	53	8
Largest scaffold size (bp)	3,549,218	3,212,662	3,597,007
Scaffold N_50_ (bp)	2,011,058	742,134	2,328,861
No. of spanned gaps	117	N/A^*b*^	0
GC content (%)	37.0	37.1	37.2
No. of genes	5,799	N/A^*b*^	5,740

^*a*^ Statistics for the new PacBio-based genome assembly are in the right-most column (GCA_030384665.1). Two other genome assemblies were available for *L. elongisporus* NRRL YB-4239 on the National Center for Biotechnology Information website (https://www.ncbi.nlm.nih.gov/genome). The RefSeq representative genome (GCA_000149685.1, ASM14968v1) was created by end-sequencing plasmid and fosmid libraries using ABI/Sanger technology. The other assembly (GCA_013620985.1; ASM1362098v1) was created from Illumina MiSeq and Oxford Nanopore MinION data.

^*b*^ N/A, not applicable; contigs were not assembled into scaffolds nor annotated.

## Data Availability

This whole-genome sequencing project was deposited in GenBank under BioProject PRJNA935742 and BioSample SAMN33325380. The genome assembly was assigned the locus prefix tag PVL30 and accession number GCA_030384665.1. Accession numbers CP128566.1 to CP128573.1 were assigned to the chromosomes described here. PacBio sequence reads were deposited into the Sequence Read Archive under accession number SRX19568455.
